# Applying a cultural multilevel selection framework to the adoption of sustainable management practices in California viticulture

**DOI:** 10.1007/s11625-017-0515-4

**Published:** 2017-12-01

**Authors:** Vicken Hillis, Adrian Bell, Jodi Brandt, Jeremy S. Brooks

**Affiliations:** 1 Human-Environment Systems, Boise State University, 1910 W University Drive, Boise, ID 83725-1075 USA; 2 Department of Anthropology, University of Utah, 270 S. 1400 E. RM 102, Salt Lake City, UT 84112 USA; 3 School of Environment and Natural Resources, The Ohio State University, 2021 Coffey Rd., Columbus, OH USA

**Keywords:** Cultural evolution, Cultural multilevel selection, Viticulture, Sustainability, California

## Abstract

In light of the ongoing environmental impacts of agriculture, understanding farmer adoption of sustainable management practices (SMPs) is an important priority. Relatively little work in agricultural adoption has explicitly examined the multilevel dynamics of adoption decision-making. Yet because many SMPs involve cooperative dilemmas—they are individually costly but provide group benefits—understanding the dynamics of both individual and group level behavioral change is critical. In this paper, we argue that cultural evolutionary theory is well suited to examining the emergence and spread of cooperative SMPs, and we illustrate this claim by applying a cultural multilevel selection (CMLS) framework to the adoption of SMPs on the part of winegrape growers in California, USA. Using survey data from over 800 winegrape growers in 3 regions, we estimate the individual-level costs and group-level benefits of 44 different SMPs. We then relate this to variation in their adoption within and between winegrape growing regions to characterize the scope for cultural group selection of the various practices. We also identify a number of mechanisms that might plausibly explain the observed patterns of variation, including various forms of cultural group selection. We highlight the added value of this perspective with respect to the established approaches and outline the data requirements for researchers to conduct similar studies in other settings. Our results underscore the potential for a cultural evolutionary perspective to shed light on the multiscale mechanisms driving adoption of SMPs and, more generally, the promise of cultural evolutionary approaches to supplement existing analytical toolkits in sustainability science.

**Electronic supplementary material:**

The online version of this article (10.1007/s11625-017-0515-4) contains supplementary material, which is available to authorized users.

## Introduction

Many agricultural systems impact the environment negatively (Gomiero et al. 2011), for example contributing to soil erosion (Montgomery 2007), biodiversity loss (Donald 2004), declining water quality or availability (Rockström et al. 2007; Moss 2008), and agrichemical pollution (Vitousek et al. 2009). Reducing these environmental impacts requires changing farmer behavior, which can be challenging when the management practices involve cooperative dilemmas—practices that produce group benefits but are costly to individual farmers. Addressing this challenge demands an understanding of the dynamics of individual and group-level behavioral change.

In this paper, we argue that a cultural evolutionary approach is well suited to examining multilevel social dynamics in agricultural systems. We establish this claim by applying this approach to existing quantitative and qualitative data about the sustainable management of winegrapes in three important winegrowing regions of California. We characterize the individual-level costs and group-level benefits of 44 different sustainable management practices (SMPs), as well as variation in their adoption within and between winegrape growing regions. In doing so, we identify the scope for cultural group selection to act on these SMPs. We identify several cultural evolutionary mechanisms that might explain these observed patterns of variation. Finally, we highlight the added value of this approach with respect to established frameworks for understanding behavioral change in agricultural settings, and we outline the data needed for researchers to quantitatively examine cultural group selection in other settings. In sum, our objective is to explicate an approach—cultural evolution—that sustainability and agricultural scientists can add to their analytical toolbox.

## Understanding behavior change in agricultural systems

One commonly used approach to examine agricultural practice adoption is found in the literature on the diffusion of innovations (Rogers 2010). Researchers in this subfield have examined the factors that cause behaviors to diffuse in a population. With over 5,200 publications, the diffusion of innovations literature is too broad and multifaceted to characterize easily, but much of the existing work has focused on how characteristics of the adopter, characteristics of the innovation, the nature of communication channels, and the structure of the social system can affect diffusion (Rogers 2010). In contrast, a primary focus of the cultural evolutionary literature has been to identify mechanisms of learning—who do people learn from in different contexts—and the group-level implications of these different mechanisms for behavioral change. Bass (1969) was one of the first to mathematically model the diffusion process, tracking frequencies of behaviors in a population over time as a function of different forms of communication, an approach consistent with much of the formal modeling in cultural evolution.

Cultural evolution can be defined as change over time in the distribution of socially transmitted traits, such as beliefs, ideas, and knowledge (Cavalli-Sforza and Feldman 1981; Boyd and Richerson 1985). This process of change is evolutionary because it arises from heritable variation and differential fitness. Individuals vary with respect to cultural traits; they hold different ideas, beliefs and knowledge. These cultural traits are transmitted among individuals via imitation, teaching and other forms of social learning. Different variants of a cultural trait spread at different rates as a function of the different learning strategies people employ. Thus, behavioral change in the population is an evolutionary process. The cultural evolutionary approach provides a formal system for tracking the spread of ideas and behaviors in a population. To date, cultural evolutionary researchers have developed a rich body of general theory and evidence identifying the learning strategies that are adaptive in varying environments and the population-level consequences of these different learning strategies (see Mesoudi 2015 for a review of the cultural evolution literature). Cultural evolutionary and diffusion of innovations approaches share a common interest in understanding the factors driving behavioral change in a population. The cultural evolutionary approach, however, is particularly well suited to situations where cross-scalar processes are important, because it naturally integrates both individual and group-level dynamics, simultaneously considering the joint role of each in agricultural behavioral change.

Cultural multilevel selection (CMLS) is a specific cultural evolutionary framework that suggests that selection can occur among individuals within a group, among groups of individuals, and among groups of groups (Wilson and Sober 1994). Just as cultural traits that confer an individual advantage are disproportionately likely to spread in a population via individual-level selection, traits that confer a group-level advantage can spread via cultural group selection. CMLS theory posits that when group selection pressures are stronger than individual selection pressures, groups of cooperative individuals can outcompete groups of selfish individuals, allowing for the spread of group-beneficial behaviors, norms, or institutions through a variety of mechanisms (Henrich 2004; Boyd and Richerson 2002).

As such, CMLS theory is particularly useful for examining behavioral dynamics in the context of cooperative dilemmas when the interests of individuals and groups are misaligned (Henrich 2004). Many SMPs are cooperative dilemmas; they involve short-term personal costs and produce long-term, shared benefits. For example, while the community overall does best when all farmers make individual sacrifices to protect local water quality, each individual farmer has a short-term incentive to free ride on the efforts of others. This is because individual farmers who free-ride avoid the costs of water quality protection while reaping the benefits generated by the effort of others. Cooperative dilemmas like water quality protection are unlikely to spread due to individual-level selection, because free-riders who forego protection do better than those who do not. If groups of farmers compete, however, then cooperative groups of farmers who successfully protect their water quality may outcompete other groups who do not. Thus, adoption of SMPs that embody cooperative dilemmas is more likely to require selection at the level of cultural groups, whereas SMPs that do not involve cooperative dilemmas will spread more easily via individual-level selection alone.

CMLS theory generates predictions about the conditions that promote or constrain the adoption of SMPs based on the degree to which they present farmers with a cooperative dilemma. Cooperative dilemmas are more likely to spread if the costs to individuals are relatively low or the benefits to groups are relatively high (both of these conditions attenuate the intensity of the cooperative dilemma), or if variation among groups is relatively high and variation within groups is relatively low, thereby increasing the scope for cultural group selection. Importantly, a number of cultural evolutionary mechanisms, such as disproportionate imitation of the majority behavior, symbolic marking of group membership, and punishment of norm violators (Henrich 2004), can serve to maintain cultural variation among groups at relatively high levels, even in the face of migration of individuals and ideas among these groups.

Despite the apparent explanatory potential of a cultural evolutionary perspective, relatively little work in agricultural decision making has explicitly adopted such an approach. Some important and recent exceptions, however, have begun to uncover the potential value of the cultural evolutionary approach for understanding behavioral change in environmental contexts more generally. For example, Wilson et al. (2013) argued that Ostrom’s core design principles for governance of natural resources can be generalized to apply to groups of any kind, and in fact are the result of cultural evolutionary processes. Ellis (2015) applied theories of sociocultural change, including cultural evolution, to an understanding of ecological process and pattern in an anthropogenic biosphere. Waring et al. (2015) explicated a CMLS framework intended for specific application to sustainability contexts, and they applied the framework to several case studies relevant to sustainability. Our case study provides an investigation of this CMLS framework, and is one of the first attempts to draw on quantitative, empirical data.

## Multilevel social structure in California viticulture

Winegrape production has substantial economic importance in California, accounting for over 600,000 acres of production (Ross 2015) and 85% of the nation’s total wine production (Department of Treasury 2017). This production comes at considerable cost to the environment with respect to soil, air and water degradation, greenhouse gas emissions, and the loss of biodiversity and wildlife habitat (Viers et al. 2013). For example, as vineyards increase in number and extent, they more frequently encroach on oak woodlands that provide important native habitat in California (Merenlender 2000). Increasingly, winegrape growers are adopting SMPs that minimize the environmental costs associated with standard methods of production (Hoffman et al. 2015).

Winegrape growers in California are embedded in multilevel, socio-economic structures that influence their decision making. They are often members of, or have contracts with, regional partnership groups and wineries. Both wineries and partnerships have formal and informal roles in determining how winegrape growers manage their production. Regional partnerships take various forms but typically involve collaborations with industry stakeholders, university extension agents, industry representatives, researchers and growers (Warner 2007). These organizations serve a variety of purposes, including marketing, development of a regional identity, creation of a formal regional brand, promoting regional tourism, and providing outreach and educational programs to growers, often focusing on sustainability. Partnerships engage with farmers through educational workshops, farm events and by disseminating information via websites and newsletters. They also operate formal certification programs that require farmers to meet certain environmental or social standards. These efforts reinforce the importance of regional identity and branding in the wine industry (Warner 2007). Local and regional groups, furthermore, are nested within broader state and national-level groups that play similar functions with respect to industry promotion and development. It is this multilevel nature of the viticulture industry—and the consequent multilevel drivers of winegrape management that makes it an interesting context in which to examine CMLS processes.

**Fig. 1:**
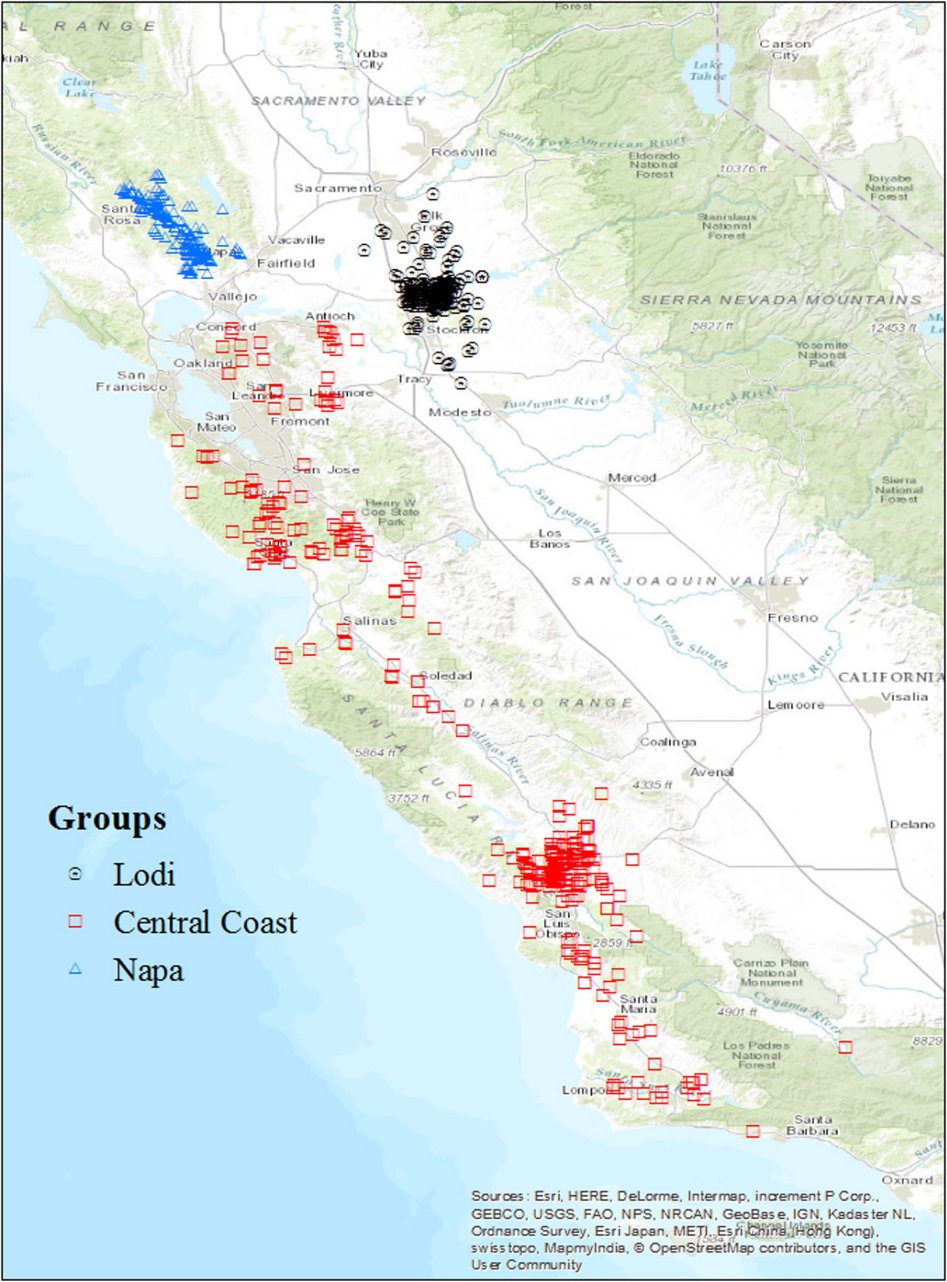
The three wine-grape growing regions and approximate locations of individual farms surveyed in each region

Our study region comprises three important winegrowing regions of California, the Napa Valley, the Central Coast, and Lodi (Fig. 1). The short descriptions provided here illustrate that regional reputation and branding are important aspects of the socioeconomic landscape of wine production in California (Warner 2007; Bruwer and Johnson 2010). The Napa valley is the foundation of the broader North Coast growing region and for several decades has been a premier winemaking region globally. It produces a wide range of varietals, and has a reputation for making high-priced wines. It is an important destination for wine tourism, particularly given its proximity to the San Francisco Bay Area. The Central Coast region is much larger than the Napa Valley, ranging from the southern California coast of Ventura county to the San Francisco Bay Area in the north. The most winegrowing areas in this region receive a substantial cooling influence from the Pacific Ocean. Chardonnay is one of the more commonly produced varietals in the Central Coast, but a wide variety of wines are produced in the region with a range of price points. The Lodi region in the northern San Joaquin valley is part of the central valley of California. Lodi is perhaps best known for its old vine Zinfandel. Vineyards in Lodi represent a wide diversity of growers and winemakers in a relatively compact area, including growers producing for large companies on large acreages at a relatively high volume and low price, often on the flat valley bottom of the central valley.

## Data sources and survey measures

To assess the scope for cultural group selection of a range of viticulture management practices, we used data from two surveys conducted in 2010–2012, as well as 16 semi-structured, in-person interviews conducted with winegrape growers in 2011. The interviews were conducted in Lodi, Napa Valley and Central Coast winegrowing regions, and asked respondents about their opinions of sustainability practices and programs. In the analysis included in this paper, we use the qualitative and ethnographic understanding generated during the interviews to inform our interpretation of the quantitative results we describe.

The first survey was conducted online in 2010 and targeted winegrape industry experts in California—university researchers, Cooperative Extension agents, trade association staff members, and vineyard management consultants. This survey provided cost and benefit measures about the 44 SMPs examined in the analyses in this paper. These SMPs consist of a range of practices considered to be sustainable forms of management based on prior research and expert consultation. The survey collected 120 responses, with an overall response rate of 43%.

The second survey involved a printed questionnaire delivered in the mail to winegrape growing decision-makers (farmers and farm managers) in the three study regions conducted in 2011 and 2012. Following Dillman (2011), this survey was delivered to all winegrape growers in relevant counties, identified using California pesticide use reports. This survey collected 822 responses, with an overall response rate of 39%. This survey asked winegrape growers whether or not they use each of the 44 practices.

While the response rates for both surveys are relatively high for agricultural surveys in the US, they do present a risk of non-response bias. We suspect that if there is a systematic bias among our respondents it may be that respondents who are interested in or supportive of environmental issues were more likely to respond. We attempted to minimize this potential effect by not framing our surveys as being related to sustainability or the environment in the introductory materials or cover page. If indeed we do have a sample biased in this way, we might observe over-reporting of adoption and group benefits, and an under-reporting of individual costs. It is not clear how this would influence within and between group variation in adoption, however, as our response rates across regions were roughly consistent. For more detailed discussions of the methods and findings of these surveys, including reporting of key respondent demographic characteristics, see Lubell et al. (2011) and Hoffman et al. (2014), respectively (both questionnaires are provided in the supplemental information).

We used responses from the online survey described above to measure the perceived costs and benefits of each of the 44 SMPs. Survey respondents ranked on 7-point Likert scales the (1) individual economic costs, (2) individual economic benefits, and (3) public environmental benefits, of each of the 44 management practices. The scale ranged from 1 to 7, with 1 representing “very inexpensive” or “no benefit” and 7 representing “very expensive” or “substantial benefit”. We calculated the net economic private costs for each practice by subtracting a respondent’s reported benefit from their cost. We used the responses to the question about public environmental benefits directly to represent a practice’s environmental benefits. For each practice, we then calculated mean private costs (or benefits, in some instances), as well as mean public benefits. We measured both the private costs and benefits.

While these measures are not in units of actual currency, they do capture the relative costs and benefits of the different practices. Furthermore, these relative ratings are consistent with those found using similar measures from an independently conducted survey by other researchers (Brodt and Thrupp 2009; Lubell et al. 2011). Practices that are costly to individuals but provide group benefits involve a cooperative dilemma. Understanding the extent to which a practice involves a cooperative dilemma is important because we expect management practices to diffuse differently as a function of whether or not they do.

## Assessing the scope for cultural group selection

To quantify patterns of adoption within and between our three regional groups, we computed cultural F_ST_ values for the 44 management practices. Cultural F_ST_ is a measure of the fraction of overall variation in a trait found between groups, in our case, the adoption of a particular management practice (for a detailed derivation see Bell et al. 2009). Larger F_ST_ values indicate that groups are very different from each other, while individuals within groups are relatively similar. Smaller F_ST_ values reflect the opposite—that individuals within groups are very different, while groups are relatively similar. Understanding this variation within and between groups is important because it relates directly to the scope for cultural group selection. When group-level variation is high (as reflected by high F_ST_ values), then there is relatively greater scope for cultural group selection. This follows from the basic logic of evolutionary theory—if there is no variation amongst units of selection, then there is nothing to select. By contrast, greater amounts of variation provide greater scope for selection. Assessing the population structure in this way is thus a first step towards understanding whether cultural group selection might be occurring in a particular case (Bell et al. 2009).

In addition to the degree of intergroup variation, the scope for group selection is also impacted by the relative costs and benefits of the practice at the individual and group levels. The greater the variation among groups in adoption of a given practice, the higher the group benefits of a practice, or the lower the individual costs, the more likely the practice is to evolve via cultural group selection. This relationship can be expressed as an inequality identifying the benefit-to-cost ratio required for a practice to spread via multilevel selection, given a particular level of variation in the practice, as follows (Bell et al. 2009; Richerson et al. 2016):

1 $$\frac{{{\text{Group benefit}}}}{{{\text{Individual cost}}}}>\frac{{1 - ~{F_{{\text{ST}}}}}}{{{F_{{\text{ST}}}}}}$$

**Fig. 2:**
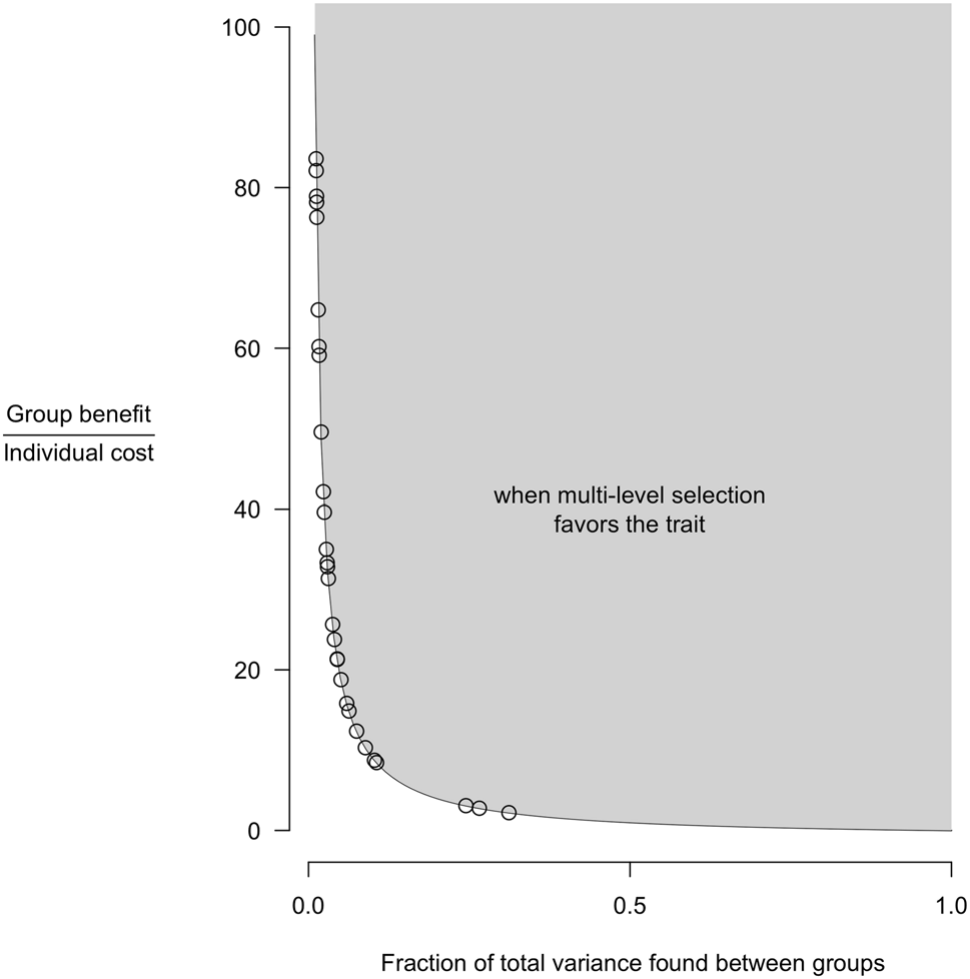
Scope for cultural group selection of the 44 different management practices. The ratio of group benefit and individual cost is plotted on the *y*-axis. The fraction of total variance found between groups (*F*_ST_) is plotted on the *x*-axis. Each point represents the hypothetical minimum benefit-to-cost ratio required for a practice to spread, given its observed level of *F*_ST_. Multilevel selection favors traits with high group benefits, low individual costs or high between-group variance, as depicted by the plotted boundary condition, following Eq. 1

The boundary condition expressed by this inequality is depicted in Fig. 2. Practices with benefit-to-cost ratios higher than those depicted by the plotted line for any given level of F_ST_ are predicted to evolve via multilevel selection (as seen in the grey-shaded region of the figure). Using the measured F_ST_ value for each practice, we display the minimum level of benefit-to-cost ratio required for each practice to evolve via multilevel selection. Ideally, we would plot the actual measured benefit-to-cost ratios for each practice, and make corresponding predictions about the spread of each practice via multilevel selection. However, because the likert scale measures are not conducive to constructing ratios, each point instead displays the minimum required benefit-to-cost ratio for the observed *F*_ST_ for that practice. As seen, the practices exhibit a range of *F*_ST_ values, and the benefit-to-cost ratio required for each of the practices to spread varies accordingly. Relatively small changes in *F*_ST_, when it is low, can correspond to large changes in benefit-to-cost ratio required for a practice to spread. Thus, many practices exhibit relatively little between-group variation and will only spread via multilevel selection if the group benefits far outweigh the individual costs. A few practices, however, exhibit enough intergroup variation that they are likely to spread even if the group benefits are not that much greater than the individual costs.

**Fig. 3:**
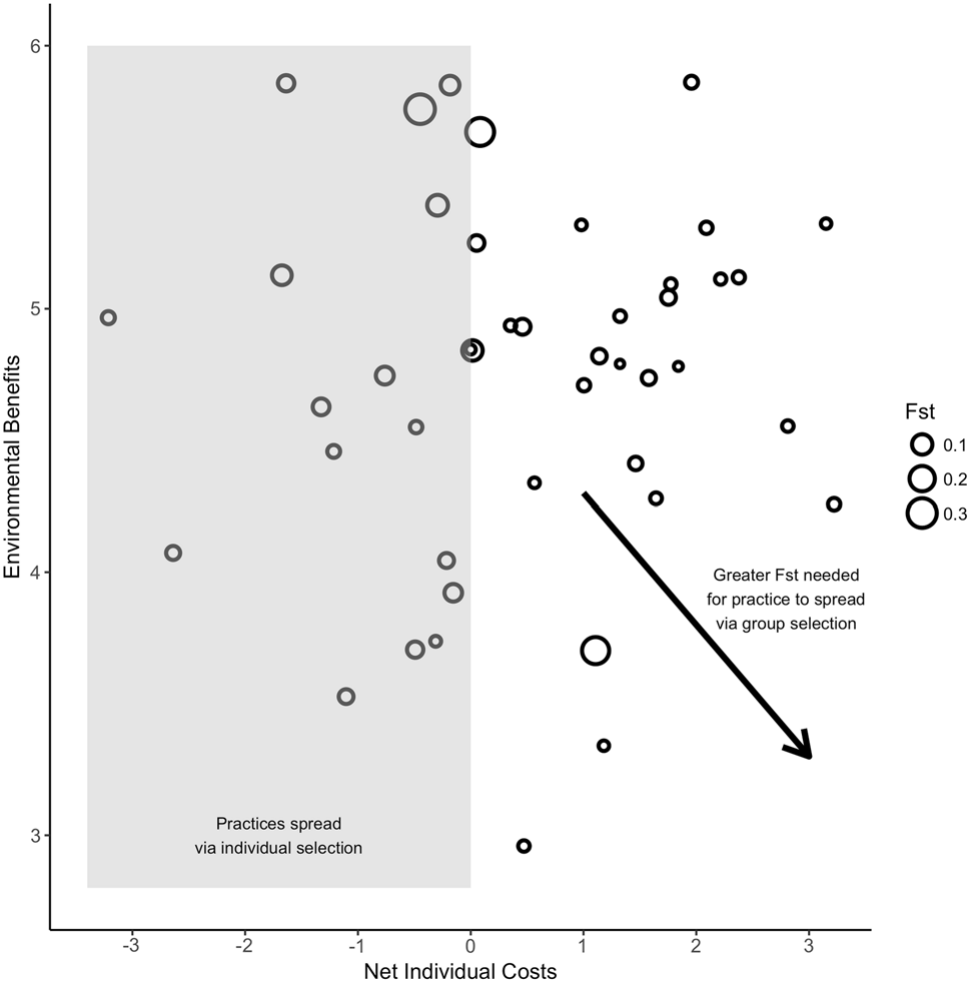
Environmental benefits as a function of the net individual costs to farmers of each practice. Each unfilled circle represents a practice, scaled in proportion to the fraction of total variance in adoption of that practice found between groups (*F*_ST_). Individually beneficial practices that might spread via individual-level selection are in the grey-shaded region. In the white-shaded region, where group selection is required for practices to spread, more intergroup variation is required as individual costs increase or group benefits decrease, in order for the practice to spread

Figure 3 displays the environmental (group) benefits as a function of the net individual costs for each of the 44 practices, as well as the fraction of total variance between groups (F_ST_) for each three-way comparison of the regions (Napa, Central Coast, and Lodi). Using the relative ratings of individual costs and group benefits, this figure conceptually represents the relationships between individual costs, group benefits, intergroup variation and selection required for the practice to diffuse. Practices on the left side of the figure (grey shading) can evolve via individual selection because they provide individual benefits. Within the grey-shaded region, intergroup variation is less important because group selection is not required for the spread of these practices. In contrast, practices on the right side of the figure (white shading) are individually costly. These practices require group selection to spread. When group selection is required, increasing intergroup variation is needed for a practice to spread as individual costs increase or group benefits decrease. This relationship is depicted by the downward sloping arrow.

## Mechanisms that maintain intergroup variation

As seen in the range of *F*_ST_ values, adoption of some practices is highly variable among individuals; adoption of others is highly variable among groups. Understanding these patterns of variation is important because practices that are highly variable across groups are those with a greater scope for cultural group selection. The cultural evolutionary perspective provides potential explanations for what might be creating these observed patterns of variation. Here, we identify a number of mechanisms that might maintain between-group variation at high levels, or dampen within-group variation, because these mechanisms enable cultural group selection. In the subsequent section, we outline a number of mechanisms of cultural group selection that might operate on this intergroup variation.

Winegrape production is famously dependent on environmental factors—any particular varietal is often limited to a specific range of environmental conditions. This idea is encapsulated in the concept of *terroir*, the natural environment in which a wine is produced, including the soil, topography and climate, and the fact that many varietals are limited to specific terroirs. These important geographic influences undoubtedly influence management decisions, either directly as a winegrape grower learns, via individual or experiential learning, what works in a particular environment, or indirectly via the transmission of information from other winegrape growers about the local environment and the practices that are most successful there.

Between-group variation can also be created and maintained via social learning, which is an important component of grower decision making (Hoffman et al. 2015). One-to-many transmission is a specific form of social learning, in which one or a few specific individuals have a disproportionate impact on the behavior of others. This type of learning can promote between-group variation and dampen within-group variation, with different groups adopting different practices (Cavalli-Sforza and Feldman 1981). Winegrape grower information-sharing networks include a few specific indviduals who are disproportionately central—this network structure facilitates one-to-many transmission (Hoffman et al. 2015). Central individuals in winegrape grower networks often have a formal extension or consulting role (Hoffman et al. 2015). Indeed, the University Extension system is effectively a formalization of one-to-many transmission, in which a handful of extension officers transmit behavioral recommendations to a population of farmers. Regional variation in management practices recommended by extension agents, therefore, can serve to promote between-group variation in management practices, thereby enabling cultural group selection across regions.

## Mechanisms of group selection

The CMLS perspective focuses our attention on competition and selection at different levels of social organization. Understanding whether individual or group-level competition is the predominant force in a particular setting changes our expectations about whether or not individuals will adopt SMPs that are cooperative dilemmas. When individuals compete, we expect individuals to engage in self-interested behavior—selfish individuals will outcompete self-sacrificing ones. When groups of individuals compete with each other, however, we expect individuals to cooperate on behalf of their respective groups—groups of self-sacrificing individuals will outcompete groups of selfish individuals.

Group-level competition in California viticulture—among wineries or even viticultural regions—can promote adoption of individually costly SMPs by winegrape growers. While group-level competition and individual-level cooperation are not necessarily explicitly related to environmental impacts, in the viticultural industry in California, they increasingly are. Competition for environmental sustainability occurs both among wineries and among viticultural regions, as embodied by the regional partnerships. Wineries can also play an important role in the emergence and spread of SMPs by requiring certified sustainable or organically grown wine grapes; some high-profile wineries already have such requirements.

Regional environmental differences are often emphasized by winegrape partnerships developing a regional identity with the aim of promoting the region’s industry. These efforts can involve branding campaigns that are sometimes even formalized into local certification programs that may or may not have sustainability components. For example, the Lodi Winegrape Commission’s website has a promotional campaign entitled “We’re LoCA”, featuring the wines of Lodi, California. These certification programs are often operated by regional partnerships, who promote adoption of SMPs to growers even if they are not taking the step of becoming formally certified. Regional branding, therefore, is often explicitly environmental. These efforts at regional branding and certification serve to homogenize growers within regions while differentiating the regions themselves, thus creating the potential for group selection.

Group-level cultural selection can occur via at least three distinct mechanisms (Henrich 2004; Richerson et al. 2016); differential imitation of more successful groups by less successful groups, differential proliferation and extinction of groups, and differential migration of individuals from less successful to more successful groups. We find substantial evidence of the disproportionate imitation of successful groups in our study system. This can occur if winegrape growers, or regional partnerships themselves, preferentially imitate growers from the more successful regions. With respect to program development, stakeholders we interviewed commonly mentioned the important role of between-region copying of program elements and materials. For example, the Lodi Winegrape Commission was the first partnership to create a self-assessment workbook that growers can use to evaluate the environmental impact of their own management practices. Subsequently, the Lodi workbook was used as a model for the creation of a similar self-assessment workbook designed by other regional and state partnerships. This is a form of between-group imitation of management practices—in this case explicitly focused on improving the environmental sustainability of the regions. Furthermore, these partnership efforts have a substantial impact on grower adoption of management practices—growers who participate in more partnership activities also adopt more SMPs (Hillis et al. under review).

Differential proliferation and extinction of groups are common in any economic enterprise where companies compete. In our study system, wineries competing against each other often aggregate groups of winegrape growers. Successful wineries will stay in business longer, potentially impacting the management practices that their contracted growers use. Unsuccessful wineries will go out of business. Their contracted growers will either also go out of business or migrate to more successful wineries. In this way, either via extinction or migration, successful wineries have an outsized influence on the management practices that winegrape growers use. To the extent that success in competition among wineries is related to reducing environmental impact, then it will promote the adoption of sustainable management practices among winegrape growers. While it is not clear that sustainably produced wine actually commands a premium in the current marketplace (Delmas and Lessem 2017), there are a number of wineries who stipulate their growers must be certified sustainable winegrape producers. As such, it appears that winery-level competition does induce adoption of SMPs among growers via the mechanisms of differential migration and extinction of growers.

Differential migration might occur when a specific region is known to be successful, thereby attracting winegrape growers from less successful regions. These new growers might adopt established management practices typical of the new region, increasing the frequency of these practices in the population overall. We do not have direct evidence of migration from less to more successful regions among winegrape growers. This mechanism might be constrained by the relatively high transaction costs involved with moving a winegrape growing operation, including the role of regional environmental variation in complicating such movements. Groups can also be conceptualized, however, as wineries rather than regions. Movement of growers among wineries as a function of winery success is common. Thus, this represents a form of migration-based cultural group selection.

## Conclusion

We examined variation in adoption of winegrape SMPs within and between regional groups in California, to assess the scope for cultural group selection in the practices. We identified a number of mechanisms of cultural group selection that are consistent with the observed patterns of variation, as well as with our qualitative understanding of the study system based on interviews and developed over years of field work. In particular, we identified a potentially important role for the differential extinction of wineries, migration of winegrape growers from less successful to more successful wineries, and imitation of management practices among winegrape growing regions in promoting the adoption of SMPs. Overall, we have explicated a case that illustrates the potential for a cultural evolutionary approach to explain adoption dynamics in the context of sustainable agriculture, but also has relevance broadly to any environmental decision-making context.

Our analysis focused primarily on the social dynamics of the system. This is, in part, because we think those dynamics are likely to be important in and of themselves (Perreault 2012), but also as a tactic for simplifying our explication of the cultural evolutionary approach. In reality, however, it is likely that spatial and environmental variation both play a role in decision making and, furthermore, that spatial and environmental factors interact with cultural evolutionary processes, both driving and responding to them. A more complete analysis would take these additional factors into account quantitatively. Some of the observed variation in practice adoption may likely be the result of environmental variation that is unrelated to mechanisms of cultural group selection. For example, the use of mechanical methods (e.g., machine-based harvesting and pruning) is an individually beneficial practice with relatively high group-level variance. This is likely because mechanical methods are preferentially used in Lodi, where the vineyards on average are flatter, bigger, and produce less expensive wines, but are inconsistent with production systems in Napa and the Central Coast where vineyards are often on more sloping terrain and production is smaller in scale.

Given that we used data that were not collected with CMLS explicitly in mind, we are unable to draw definitive conclusions about whether and to what extent cultural group selection is occurring in our system. While the observed patterns of variation are consistent with cultural group selection, they do not exclude competing explanations. In particular, the data we used here lack the temporal and individual-level variation with respect to costs and benefits required to explicitly identify whether CMLS is driving behavioral change. With a research design and data collection specifically designed for CMLS analysis (see Kline et al. 2017), applying a cultural evolutionary perspective has the potential to uncover the individual and group-level dynamics driving sustainability in a particular system of interest. The data required to conduct a quantitative test of CMLS are substantial, but still feasible, and include variation in adoption of a trait (or practice) over time, the individual and group-level costs and benefits associated with the trait, and the relationship between the adoption of the trait and individual- and group-level economic or environmental outcomes.

This analysis serves as an important preliminary example of applying the logic of cultural evolution in the context of environmental sustainability and, in particular, the adoption of sustainable agricultural practices. The cultural evolutionary perspective highlights the multilevel processes driving grower adoption of different management practices, and subsequently the environmental impacts of agricultural management. As the cultural evolutionary approach matures and becomes more widely applied, we argue that it can productively expand the range of analytical tools available to sustainability scientists.

## Electronic supplementary material

Below is the link to the electronic supplementary material.

Supplementary material 1 (PDF 832 KB)

Supplementary material 2 (PDF 258 KB)
