# Supplementary material for: Applying a cultural multilevel selection framework to the adoption of sustainable management practices in California viticulture
Source: Sustain Sci. 2017 Dec 1;13(1):71–80. doi: 10.1007/s11625-017-0515-4 (PMC6086253; doi:10.1007/s11625-017-0515-4)

2011 Lodi Winegrape Grower Survey

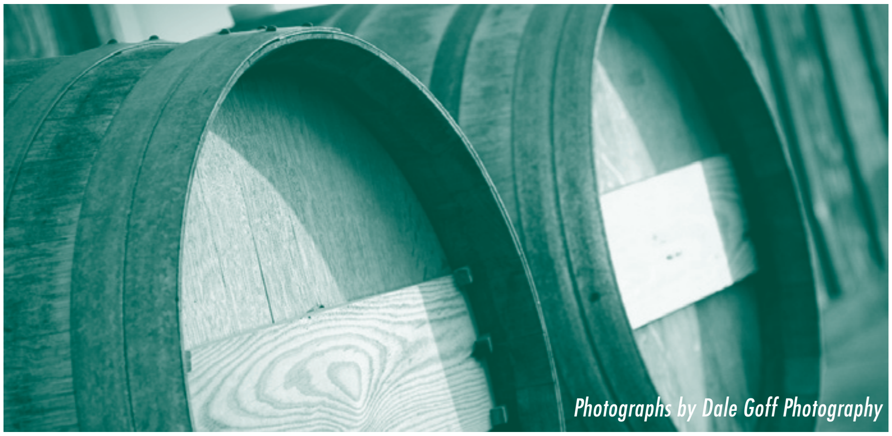

Photographs by Dale Goff Photography

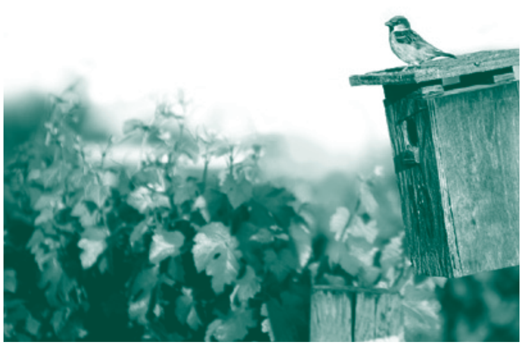

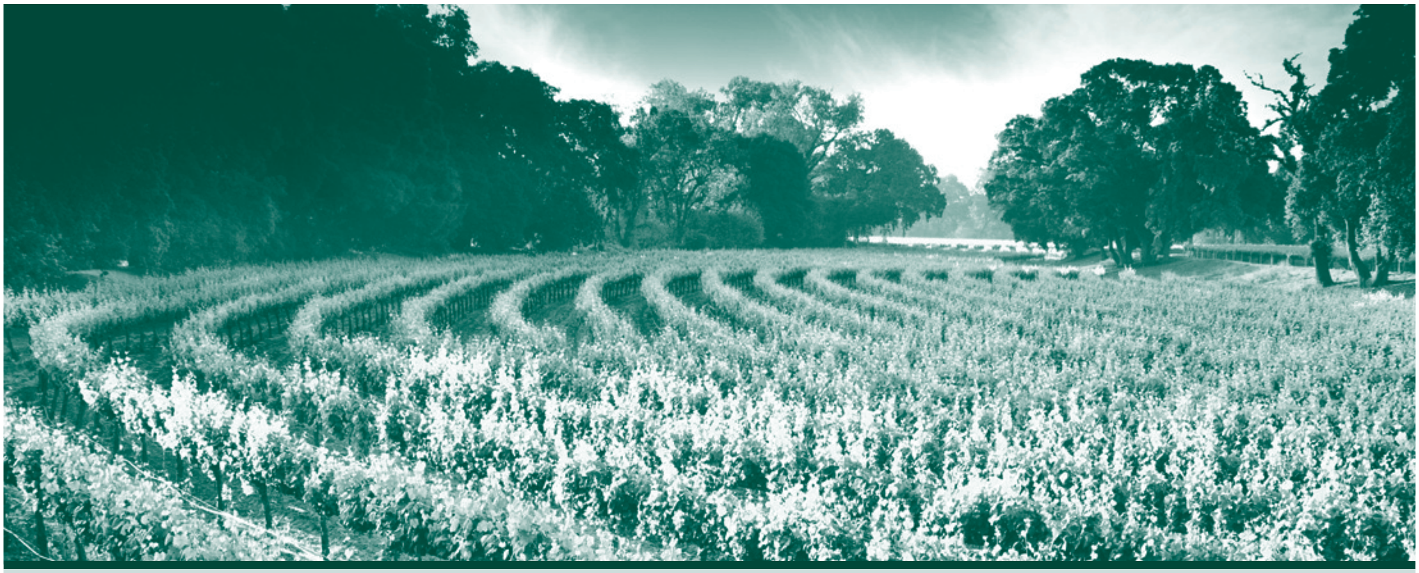

Research Project Director:

Mark Lubell, Professor

University of California at Davis • (530) 752-5880 • mnlubell@ucdavis.edu

Research Project Sponsors:

UC DAVIS
UNIVERSITY OF CALIFORNIA

LODI
WINEGRAPE
COMMISSION

Please return your complete questionnaire in the enclosed, pre-paid envelope to:

Dr. Mark Lubell

DESP
One Shields Avenue
University of California Davis
Davis, CA 95616

2011 LODI WINEGRAPE GROWER SURVEY

Respondent Confirmation

This survey is intended for people with primary decision responsibility for viticulture management in the winegrape growing enterprise indicated on the cover letter. If you are not the person with primary decision responsibility for viticulture management in the winegrape enterprise, please give this survey to the person with primary responsibility.

Are you the individual addressed in the cover letter (please mark one)?

☐ **Yes** ☐ **No**

If you were given this survey by the person addressed in the cover letter, please briefly explain your relationship. For example, you might be the vineyard manager for a landowner.

____

____

____

____

____

____

____

____

If your winegrape growing enterprise is associated with multiple viticulture managers and you need more surveys, or if you have received multiple surveys through the mail or from other people, or if you have any other questions, please contact principal investigator Mark Lubell using the information below:

Mark Lubell, Ph.D.
University of California, Davis
vitresearch@ucdavis.edu
(530) 752-5880

Instructions

This is the third installment of the Lodi Winegrape Grower Survey. The first and second surveys, administered in 1998 and 2003, collected important information used by the Lodi Winegrape Commission (LWC) to improve outreach strategies and services to Lodi winegrape growers. This survey allows you to express your views and opinions about viticulture management and outreach programs. If you decide to complete this survey it will take approximately 30 minutes of your time. **All data from this survey will be kept anonymous. Your privacy is our priority.** Thank you in advance for helping to improve Lodi viticulture and California agriculture.

SECTION A:
Viticulture Management Practices

For the following list of management practices, please indicate whether you regularly use each practice, whether you tried the practice in the past but later discontinued it, or whether you never used the practice at all. Regularly used practices might be implemented every year, but also could be less frequent if appropriate. For each practice that you regularly use or tried, please estimate what year you first started using it.

	Current and Past Use of Practices (Please ✓ the best answer.)			*Year First Used* Enter year; e.g., 1970
**Insect and Mite Management**	**Regularly Use**	**Tried & Discontinued**	**Never Used**	
Maintain written monitoring records for pests	☐	☐	☐	Year ______
Spot spraying instead of treating entire vineyard	☐	☐	☐	Year ______
Reduced pesticide application rates (using conventional equipment)	☐	☐	☐	Year ______
Pheromones for pest mating disruption	☐	☐	☐	Year ______
Cover crops (planted or resident) for natural enemy refuge	☐	☐	☐	Year ______
Release beneficial/natural predators or parasites	☐	☐	☐	Year ______
Maintain written monitoring records for natural enemies	☐	☐	☐	Year ______
Dust reduction on roads	☐	☐	☐	Year ______
Dust reduction with cover crops	☐	☐	☐	Year ______
**Disease Management**				
Using computer disease forecasting model (e.g., Powdery Mildew Model)	☐	☐	☐	Year ______
Irrigation management to reduce disease	☐	☐	☐	Year ______
Leaf pulling	☐	☐	☐	Year ______
Remove diseased wood and fruit and clean berms	☐	☐	☐	Year ______
Remove infected vines	☐	☐	☐	Year ______
Manage pruning decisions (e.g., timing and sanitation) to reduce disease spread	☐	☐	☐	Year ______
**Weed Management**				
Written monitoring records and need-based spraying	☐	☐	☐	Year ______
Mechanical weed management	☐	☐	☐	Year ______
Use only contact herbicides/no pre-emergents	☐	☐	☐	Year ______
Narrowing the width of the treated strip	☐	☐	☐	Year ______
Shielded sprayer to minimize drift	☐	☐	☐	Year ______
Reduced herbicide application rates using conventional equipment	☐	☐	☐	Year ______
**Water and Soil Management**				
Use ET-based methods to determine when to irrigate	☐	☐	☐	Year ______
Rely on visual observations to determine when to irrigate	☐	☐	☐	Year ______
Use regulated deficit irrigation (RDI) methods	☐	☐	☐	Year ______
Soil moisture tests to track water availability	☐	☐	☐	Year ______

Measure plant water stress (e.g., pressure bomb)	☐	☐	☐	Year ________
Mapping for soil water holding capacity	☐	☐	☐	Year ________
Use of vegetative filter strips to reduce runoff into waterways	☐	☐	☐	Year ________
Written erosion control plan	☐	☐	☐	Year ________
Diversion structures (e.g., straw bales, water bars) to divert or contain seasonal water flows	☐	☐	☐	Year ________
Soil tests for nutrient content, pH, electrical conductivity (EC), and toxicity	☐	☐	☐	Year ________

Other Vineyard and Operation Management Practices

Use of compost in vineyards	☐	☐	☐	Year ________
Owl boxes/perches for birds of prey	☐	☐	☐	Year ________
Develop a written company "sustainability" plan	☐	☐	☐	Year ________
Develop a written human resource plan	☐	☐	☐	Year ________
Develop a written plan to transition the operation to the next generation, or a "succession" plan	☐	☐	☐	Year ________
Vineyard management implemented to achieve overall "vine balance"	☐	☐	☐	Year ________
Monitor and record canopy microclimate throughout growing season	☐	☐	☐	Year ________
Third-party certification for "sustainable" or "green" viticulture	☐	☐	☐	Year ________
Use of alternative electricity sources such as wind or solar	☐	☐	☐	Year ________
Use of alternative fuels such as bio-diesel, propane, natural gas, or methane	☐	☐	☐	Year ________
Disposal of removed vines by means other than burning	☐	☐	☐	Year ________
Monitor and record total energy (fuel and electricity) use	☐	☐	☐	Year ________
Mechanical methods for major viticultural activities (i.e., mechanical pruning/harvesting)	☐	☐	☐	Year ________

We are interested in how you prioritize different goals in your viticulture management decisions. For each goal listed below, would you say it is never, sometimes, often, or always a major priority in your viticulture management decisions? Please mark the best answer.

How often is each goal listed below a major priority in your viticulture management decisions? (Please ✓ the best answer)

Management Goal	Never	Sometimes	Often	Always
Profitability of your operation	☐	☐	☐	☐
Wildlife habitat restoration	☐	☐	☐	☐
Employee well-being	☐	☐	☐	☐
Public health and safety	☐	☐	☐	☐
Local community quality of life	☐	☐	☐	☐
Winegrape quality	☐	☐	☐	☐
Winegrape quantity/yield	☐	☐	☐	☐
Water quality	☐	☐	☐	☐
Meet winery expectations	☐	☐	☐	☐
Ecological biodiversity	☐	☐	☐	☐
Generational succession of farm enterprise ownership	☐	☐	☐	☐
Regional reputation	☐	☐	☐	☐
Meet government regulations	☐	☐	☐	☐
Water availability	☐	☐	☐	☐

SECTION B:
Viticulture Outreach and Education Programs

The Lodi Winegrape Commission (LWC) and other organizations offer different types of outreach activities and programs. Please indicate whether or not you have participated in the following outreach activities and programs in the past five years. Mark "Never heard of the activity" if appropriate.

Have you participated in this activity in the last five years? (Please ✓ the best answer.)

	Yes	No	Never heard of the activity
Attended field meetings	☐	☐	☐
Attended breakfast meetings	☐	☐	☐
Read LWC newsletter	☐	☐	☐
Talked with LWC staff	☐	☐	☐
Completed Lodi Rules Certification	☐	☐	☐
Completed Lodi Winegrowers Workbook self-assessment	☐	☐	☐
Attended Lodi Winegrowers Workbook workshops	☐	☐	☐
Accessed LWC internet resources	☐	☐	☐
Attended Grape Day	☐	☐	☐
Completed the state-wide workbook (Code of Sustainable Winegrowing) self-assessment	☐	☐	☐

In your opinion, how successful has the Lodi Sustainable Winegrowing Program been at achieving the following goals? Please base your answer on the activities in which you have participated, or if you have not directly participated, based on what you have learned from other sources. For each goal, do you think the program has been very unsuccessful, somewhat unsuccessful, neutral, somewhat successful, or very successful in meeting that goal? Answer "Don't Know" only if you have not heard enough about the program to provide an opinion for a given goal. ***(Please ✓ the best answer.)***

	Very Unsuccessful	Somewhat Unsuccessful	Neutral	Somewhat Successful	Very Successful	Don't Know
Improve economic returns to growers	☐	☐	☐	☐	☐	☐
Reduce input costs	☐	☐	☐	☐	☐	☐
Improve consumer perceptions of the region	☐	☐	☐	☐	☐	☐
Improve relationships between viticulture industry and regulatory agencies	☐	☐	☐	☐	☐	☐
Improve vineyard yield	☐	☐	☐	☐	☐	☐
Improve well-being of farm laborers	☐	☐	☐	☐	☐	☐
Reduce labor costs	☐	☐	☐	☐	☐	☐
Improve winegrape quality	☐	☐	☐	☐	☐	☐
Improve wildlife habitat	☐	☐	☐	☐	☐	☐
Reduce health risks to the community	☐	☐	☐	☐	☐	☐
Improve biodiversity	☐	☐	☐	☐	☐	☐
Reduce environmental risks	☐	☐	☐	☐	☐	☐
Reduce quantity of recordkeeping necessary to participate	☐	☐	☐	☐	☐	☐
Reduce amount of time needed to manage vineyards	☐	☐	☐	☐	☐	☐
Reduce uncertainty about vineyard productivity	☐	☐	☐	☐	☐	☐
Other (please list)	☐	☐	☐	☐	☐	☐

In general, how do you rank your level of support for Lodi's Sustainable Winegrowing Program? (Please ✓ the best answer.)

Strongly Oppose	Somewhat Oppose	Neutral	Somewhat Support	Strongly Support	Don't Know
☐	☐	☐	☐	☐	☐

In general, how do you rank your level of support for the Lodi Rules for Sustainable Winegrowing certification program? (Please ✓ the best answer.)

Strongly Oppose	Somewhat Oppose	Neutral	Somewhat Support	Strongly Support	Don't Know
☐	☐	☐	☐	☐	☐

In general, how do you rank your level of support for the state-wide Sustainable Winegrowing Program sponsored by the California Sustainable Winegrowing Alliance? (Please ✓ the best answer.)

Strongly Oppose	Somewhat Oppose	Neutral	Somewhat Support	Strongly Support	Don't Know
☐	☐	☐	☐	☐	☐

In general, how do you rank your level of support for the state-wide Certified California Sustainable Winegrowing certification program sponsored by the California Sustainable Winegrowing Alliance? (Please ✓ the best answer.)

Strongly Oppose	Somewhat Oppose	Neutral	Somewhat Support	Strongly Support	Don't Know
☐	☐	☐	☐	☐	☐

"Sustainable" means different things to different people. How do you define sustainable agriculture?
Please use the space below to define sustainable agriculture in your own words.

SECTION C:
Learning about Viticulture Management

Winegrape growers learn about viticulture management from a variety of sources, including outreach organizations, published materials, personal relationships, and personal experience. If you have used the resource, did you think it was not useful, somewhat useful, or very useful? If you have never used the listed resource, please mark "Never Used".

Please ✓ only one answer per source.

Organizations	Never used	Not Useful	Somewhat	Very Useful
State or County Farm Bureau	☐	☐	☐	☐
County Farm Advisors	☐	☐	☐	☐
County Agricultural Commissioner	☐	☐	☐	☐
Resource Conservation District	☐	☐	☐	☐
Regional Water Resources Control Board	☐	☐	☐	☐
Local Grape Grower Association	☐	☐	☐	☐
State Grape Grower Association	☐	☐	☐	☐
Winegrape Sustainability Programs	☐	☐	☐	☐
Natural Resources Conservation Service	☐	☐	☐	☐
Trade association conferences/symposiums	☐	☐	☐	☐
**Published Materials**				
Trade journals	☐	☐	☐	☐
Internet resources	☐	☐	☐	☐
Viticulture textbooks or other reference books	☐	☐	☐	☐
University publications	☐	☐	☐	☐
Newspapers	☐	☐	☐	☐
Lodi Winegrowers Workbook	☐	☐	☐	☐
California Code of Sustainable Winegrowing Workbook	☐	☐	☐	☐
**Personal Relationships**				
Winery personnel	☐	☐	☐	☐
Your field crew	☐	☐	☐	☐
Pest Control Advisors	☐	☐	☐	☐
Winegrape growers who are not your relatives	☐	☐	☐	☐
Viticulture consultant	☐	☐	☐	☐
Winegrape growers who are in your family	☐	☐	☐	☐
**Personal Experience**				
Trial and error with vineyard practices	☐	☐	☐	☐
Observations of your own vineyard conditions	☐	☐	☐	☐
Observations of other growers' vineyard conditions	☐	☐	☐	☐
Written records of vineyard inputs and performance	☐	☐	☐	☐
Field trials conducted on your vineyards	☐	☐	☐	☐
Field trials conducted by others	☐	☐	☐	☐
**Other important sources of information**				
(Please list)	☐	☐	☐	☐
(Please list)	☐	☐	☐	☐

SECTION D:
Communication about Viticulture Management

We would like to know about the people you communicate with regarding viticulture management, including other growers and other types of individuals with expertise in viticulture management. In the boxes below, please list up to four other growers, and four other types of individuals, with whom you communicated and shared knowledge about viticulture management in the past year.

These questions are important for understanding how growers communicate and learn from each other about viticulture management. *We value your privacy and are legally required to protect it. Identity is confidential, your answers will be used anonymously, and no personal names will* ***ever*** *be publicly released.*

Please list the names of up to four growers with whom you communicate about viticulture management. List in order of frequency of communication, with most frequent first.

Grower 1 Name ____________________

Grower 2 Name ____________________

Grower 3 Name ____________________

Grower 4 Name____________________

Please list the names of up to four other types of individuals with whom you communicate about viticulture management. Please write down their type of job (for example: PCA, Extension Specialist, Viticulture Consultant, or any others). List in order of frequency of communication, with most frequent first.

Other Individual 1 Name ____________________

Type of Job ____________________

Other Individual 2 Name ____________________

Type of Job ____________________

Other Individual 3 Name ____________________

Type of Job ____________________

Other Individual 4 Name ____________________

Type of Job ____________________

SECTION E:
Grower and Winegrape Enterprise Information

What is your age? Please check one.

☐ Younger than 25 ☐ 25-34 ☐ 35-44 ☐ 45-54 ☐ 55-64 ☐ Older than 65

What level of formal education have you completed? Please check one.

☐ High school, no degree earned ☐ High school, degree earned ☐ College, no degree earned
☐ College, degree earned ☐ Post college, no degree ☐ Advanced degree (e.g. MS, PhD, JD)

Have you received any specialized training in viticulture or agriculture? Please check all that apply.

☐ Pest Control Advisor ☐ Viticulture degree ☐ Enology degree Other ____________________

How many years have you been working in viticulture? ______________________________

How many generations has your family been involved in agriculture? Please check one.

☐ One generation (Immediate family) ☐ Two generations (Parents) ☐ Three generations (Grandparents)
☐ Four generations (Great grandparents) ☐ Five generations (Great, great grandparents)
☐ More than five generations

Do you consider yourself a full-time or part-time winegrape grower? Please check one.

☐ Full-time ☐ Part-Time

Winegrape growing enterprises may have complex ownership and management structures. Please answer the following questions about the ownership status of the land you manage for winegrapes, acres under different certification, number of varietals, and diversity of your operation in 2010. *We value your privacy and are legally required to protect it. Your answers are confidential, will be used anonymously, and no personal names will **ever** be publicly released.*

Ownership	Enter a number
Number of acres of winegrapes managed in 2010 in Lodi	________ Acres
Number of acres of winegrapes managed in 2010 in total (all regions)	________ Acres
Number of acres owned of total in all regions	________ Acres
Number of acres leased of total in all regions	________ Acres
Number of acres managed as hired consultant/contractor of total in all regions	________ Acres
**Certifications**	
Of the total acres you manage, how many are certified organic?	________ Acres
In what year did you begin organic certification?	________ Year
Of the total acres you manage, how many are third-party certified sustainable?	________ Acres
In what year did you begin third-party sustainable certification?	________ Year
**Operation Diversity**	
Approximately how many counties do you manage winegrapes in?	______ Counties
Approximately how many individual ranches do you manage?	______ Ranches

Winegrape growing enterprises have a diversity of marketing and sales strategies. Please estimate the number of wineries your enterprise sells grapes to for each scale of winery below. If you are a hired employee, please answer for the winegrape enterprise you are employed by.

**Scale of winery**	Number of wineries at each scale you sell winegrapes to. Please enter a number.
Less than 2000 cases per year	
2,000-9,999 cases per year	
10,000-49,999 cases per year	
50,000-99,999 cases per year	
100,000-499,999 cases per year	
500,000-999,999 cases per year	
Greater than 1 million cases per year	

If you grow certified sustainable winegrapes, do you sell your winegrapes to a winery that offers a price premium for certification? Please check one.

☐ Yes ☐ No

If you grow certified sustainable winegrapes, is your relationship with wineries stronger and more secure as a result of certification? Please check one.

☐ Yes ☐ No

Do any of the viticulture enterprises you manage or own also operate a winery (vertical integration)? Please check one.

☐ Yes ☐ No ☐ Not Applicable

Do you consider your viticulture enterprise to be economically secure? Please check one.

☐ Yes ☐ No

Please estimate your vineyard operation's annual gross income from winegrape growing before expenses and taxes. Please check one. *We value your privacy and are legally required to protect it. Your answers are confidential and only summary and anonymous results will be reported.*

☐ Less than $50,000 ☐ $50,000 - $99,999 ☐ $100,000 - $499,999
☐ $500,000 - $999,999 ☐ $1,000,000 - $4,999,999 ☐ Greater than $5,000,000

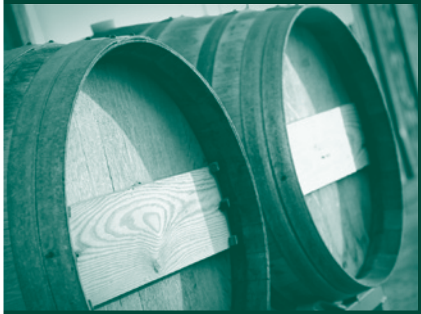
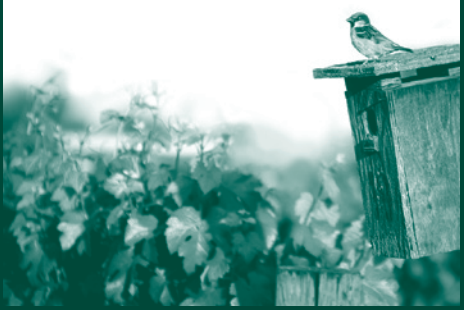
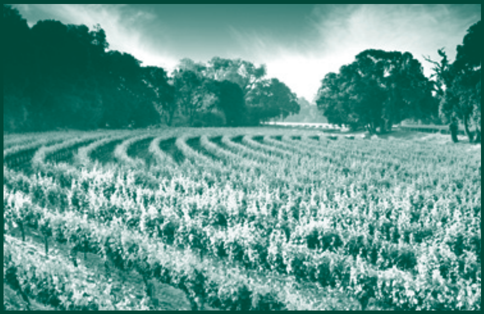

Thank you for your Help!

Please use the space below if there is anything you would like to tell us about.

If you would like to receive a summary report of this survey, please contact:

Dr. Mark Lubell
DESP
One Shields Avenue, University of California, Davis, CA 95616
(530) 752-5880 mnlubell@ucdavis.edu

CODE

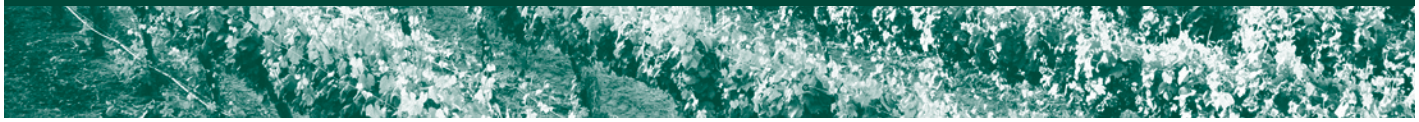

Supplement: Supplementary file 1 — Supplementary material 1 (PDF 832 KB) [file 11625_2017_515_MOESM1_ESM.pdf]
