# Supplementary material for: Applying a cultural multilevel selection framework to the adoption of sustainable management practices in California viticulture
Source: Sustain Sci. 2017 Dec 1;13(1):71–80. doi: 10.1007/s11625-017-0515-4 (PMC6086253; doi:10.1007/s11625-017-0515-4)
Supplement: Supplementary file 2 — Supplementary material 2 (PDF 258 KB) [file 11625_2017_515_MOESM2_ESM.pdf]

Cal Sust Vit - Post Advisor Comments

California Viticultural Practices: Introduction

Purpose:
You are being asked to participate in a research study investigating decision-making in California viticulture. We hope to learn about your views on the costs and benefits to growers of different practices, as well as the benefits to the community at large. This study is sponsored by UC Davis and funded by the National Science Foundation. If you decide to volunteer, you will complete the following survey, lasting approximately 20 minutes. You can exit the survey and return to it at any time and any partial progress will be saved automatically. Once you have completed the survey you will not be able to return to the survey to modify your answers. All data from this study will be kept anonymous. We hope to use the results of this survey and related research to inform and improve viticultural practices and agricultural policy in California. **Thanks in advance for helping to make California agriculture more effective.**

Participant Information Statement:
Approximately 250 other individuals will also be filling out the same survey. There is minimal risk to you of participating in this survey. It is possible that you may not benefit directly by participating in this study. Data from this study will be kept anonymous. Research documents will be kept confidential in accordance with the law and University policies. You will not be compensated for being in this study. There is no cost to you beyond the time and effort required to complete the procedure described above. You may refuse to participate in this study. You may change your mind about being in the study and quit after the study has started. If you have any questions about this research project please contact Mark Lubell who will answer them.

Mark Lubell, Ph.D.
Department of Environmental Science and Policy
University of California, Davis
vitresearch@ucdavis.edu
530-752-5880

Cal Sust Vit - Post Advisor Comments

Background Information

Please answer the following questions about your background and experience in the field of viticulture.

What is your official job title?

Which of the following job categories best describes your position? (Check only one.)

- Cooperative Extension
- Vineyard Manager/Consultant
- University Faculty/Staff
- Viticulture Producer Group Staff
- Resource Conservation District Staff
- Government Agency Staff
- Other (please specify)

Which of the following best describes your professional expertise? (Check all that apply.)

- Plant Pathology
- Weed Science
- Fundraising
- Horticulture/Small farms
- Education/Outreach
- Plant Physiology
- Air/Water Quality Management
- Media/Marketing
- Administration
- Irrigation Specialist
- Enology
- Soil Science
- Genetics
- Viticulture
- Pest Management
- Other (please specify)

How many years of experience do you have in viticulture?

Years

How involved are you with on-the-ground decisions about viticultural management? (Check only one.)

	Not involved at all	Advisory capacity only	Occasional on-the-ground decisions	Frequent on-the-ground decisions
Degree of involvement				

Cal Sust Vit - Post Advisor Comments

In your current position, you may have engaged in several types of activities where you share information with winegrape growers and the viticultural industry. Which of the following activities have you participated in? (Check all that apply.)

- Informal communication with growers
- Industry publications
- Newsletters
- On-farm field meetings
- Workbook development
- Third-party certification development
- Grower meetings
- "How-to" manuals for viticulture practices
- University research and publications
- Viticulture conferences
- Other (please specify)

Cal Sust Vit - Post Advisor Comments

Geographic Region and American Viticultural Area

Please answer the following questions about the geographic region/s in which you work most often.

From the dropdown menus, select the county or counties in which you work the most often. Select UP TO five counties, with the one in which you work the most often listed first.

	County in which you work most often	Other county	Other county	Other county	Other county
County					

Please identify up to five American Viticultural Areas (AVA) or appellations in which you work the most often. List the AVA in which you work the most often in the first box. Please list the AVAs at the smallest level of geographic resolution you know, or "sub-AVA" level. For example, the Napa Valley AVA encompasses the Rutherford "sub-AVA" among others. Click here for a list of AVAs in California.

AVA in which you work most often

other AVA

other AVA

other AVA

other AVA

From the following range of sizes of viticultural operations, select the ones in which you have experience working in. (Check all that apply.)

	0-50 acres	51-150 acres	151-250 acres	251-350 acres	351-450 acres	451-550 acres	551-650 acres	651-750 acres	751-850 acres	851-950 acres	greater than 950 acres
Viticultural Operation Size											

From the following range of sizes of viticultural operations, select the SINGLE size in which you have the MOST experience. (Check only one.)

	0-50 acres	51-150 acres	151-250 acres	251-350 acres	351-450 acres	451-550 acres	551-650 acres	651-750 acres	751-850 acres	851-950 acres	greater than 950 acres
Viticultural Operation Size											

Cal Sust Vit - Post Advisor Comments

Do you have enough experience with viticultural practices in your region to provide your views about their costs, benefits, and effectiveness? Please answer the question and click next to proceed to the subsequent section.

- Yes
- No

Cal Sust Vit - Post Advisor Comments

Costs and Benefits: Insect and Mite Management

The following questions ask you to estimate the costs and benefits to growers of various practices. When answering, consider the costs and benefits that would accrue to a viticultural operation in the geographic region you have the most experience in, and of the size you have the most experience working with. Assume that the practices are being correctly implemented.

First, read the three questions below. Then, for each practice, select an answer to each corresponding question using the dropdown menus.

1) How expensive is it to implement each of the following practices? For each practice, please select your answer on a scale from 1 to 7, where a score of 1 = "Very Inexpensive" and 7 = "Very Expensive". Select "Don't Know" if you're not familiar enough with the practice to accurately estimate its costs.

2) To what extent does implementing each of the following practices produce economic benefits to the grower? Examples of economic benefits might include, but are not limited to, improvements in winegrape quality, reductions in input costs, and improved vineyard health. For each practice, please select your answer on a scale from 1 to 7, where a score of 1 = "No Benefits" and 7 = "Substantial Benefits". Select "Don’t Know" if you’re not familiar enough with the given practice to estimate its benefits.

3) To what extent does implementing each of the following practices improve general environmental quality? Examples of environmental benefits might include, but are not limited to, improvements in water quality, water-use efficiency, wildlife habitat, and soil erosion control. For each practice, select your answer on a scale from 1 to 7, where a score of 1 = "No Benefits" and 7 = "Substantial Benefits". Select "Don’t Know" if you’re not familiar enough with the practice to estimate its benefits.

Insect and Mite Management

	1) Economic expense	2) Economic benefits	3) Environmental benefits
Maintaining written monitoring records for pests			
Spot spraying instead of treating entire vineyard			
Reduced pesticide application rates (using conventional equipment)			
Pheromones for pest mating disruption			
Cover crops (planted or resident) for natural enemy refuge			
Release beneficials/natural predators			

Cal Sust Vit - Post Advisor Comments

Maintain written monitoring records for natural predators			
Dust reduction on roads			
Dust reduction with cover crops (planted or resident)			

Cal Sust Vit - Post Advisor Comments

Costs and Benefits: Disease Management

The following questions ask you to estimate the costs and benefits to growers of various practices. When answering, consider the costs and benefits that would accrue to a viticultural operation in the geographic region you have the most experience in, and of the size you have the most experience working with. Assume that the practices are being correctly implemented.

First, read the three questions below. Then, for each practice, select an answer to each corresponding question using the dropdown menus.

1) How expensive is it to implement each of the following practices? For each practice, please select your answer on a scale from 1 to 7, where a score of 1 = "Very Inexpensive" and 7 = "Very Expensive". Select "Don't Know" if you're not familiar enough with the practice to accurately estimate its costs.

2) To what extent does implementing each of the following practices produce economic benefits to the grower? Examples of economic benefits might include, but are not limited to, improvements in winegrape quality, reductions in input costs, and improved vineyard health. For each practice, please select your answer on a scale from 1 to 7, where a score of 1 = "No Benefits" and 7 = "Substantial Benefits". Select "Don't Know" if you're not familiar enough with the given practice to estimate its benefits.

3) To what extent does implementing each of the following practices improve general environmental quality? Examples of environmental benefits might include, but are not limited to, improvements in water quality, water-use efficiency, wildlife habitat, and soil erosion control. For each practice, select your answer on a scale from 1 to 7, where a score of 1 = "No Benefits" and 7 = "Substantial Benefits". Select "Don't Know" if you're not familiar enough with the practice to estimate its benefits.

Disease Management

	1) Economic expense	2) Economic benefits	3) Environmental benefits
Using computer disease forecasting model (e.g., Powdery Mildew Model)			
Irrigation management to reduce disease			
Leaf pulling			
Remove diseased wood and fruit and clean berms			
Remove infected vines			
Manage pruning decisions (e.g. timing and sanitation) to reduce disease spread			

Cal Sust Vit - Post Advisor Comments

Costs and Benefits: Weed Management

The following questions ask you to estimate the costs and benefits to growers of various practices. When answering, consider the costs and benefits that would accrue to a viticultural operation in the geographic region you have the most experience in, and of the size you have the most experience working with. Assume that the practices are being correctly implemented.

First, read the three questions below. Then, for each practice, select an answer to each corresponding question using the dropdown menus.

1) How expensive is it to implement each of the following practices? For each practice, please select your answer on a scale from 1 to 7, where a score of 1 = "Very Inexpensive" and 7 = "Very Expensive". Select "Don't Know" if you're not familiar enough with the practice to accurately estimate its costs.

2) To what extent does implementing each of the following practices produce economic benefits to the grower? Examples of economic benefits might include, but are not limited to, improvements in winegrape quality, reductions in input costs, and improved vineyard health. For each practice, please select your answer on a scale from 1 to 7, where a score of 1 = "No Benefits" and 7 = "Substantial Benefits". Select "Don't Know" if you're not familiar enough with the given practice to estimate its benefits.

3) To what extent does implementing each of the following practices improve general environmental quality? Examples of environmental benefits might include, but are not limited to, improvements in water quality, water-use efficiency, wildlife habitat, and soil erosion control. For each practice, select your answer on a scale from 1 to 7, where a score of 1 = "No Benefits" and 7 = "Substantial Benefits". Select "Don't Know" if you're not familiar enough with the practice to estimate its benefits.

Weed Management

	1) Economic expense	2) Economic benefits	3) Environmental benefits
Written monitoring records and need-based spraying			
Mechanical weed management			
Use only contact herbicides/no pre-emergents			
Narrowing the width of the treated strip			
Shielded sprayer to minimize drift			
Reduced herbicide application rates using conventional equipment			

Cal Sust Vit - Post Advisor Comments

Costs and Benefits: Water and Soil Management

The following questions ask you to estimate the costs and benefits to growers of various practices. When answering, consider the costs and benefits that would accrue to a viticultural operation in the geographic region you have the most experience in, and of the size you have the most experience working with. Assume that the practices are being correctly implemented.

First, read the three questions below. Then, for each practice, select an answer to each corresponding question using the dropdown menus.

1) How expensive is it to implement each of the following practices? For each practice, please select your answer on a scale from 1 to 7, where a score of 1 = "Very Inexpensive" and 7 = "Very Expensive". Select "Don't Know" if you're not familiar enough with the practice to accurately estimate its costs.

2) To what extent does implementing each of the following practices produce economic benefits to the grower? Examples of economic benefits might include, but are not limited to, improvements in winegrape quality, reductions in input costs, and improved vineyard health. For each practice, please select your answer on a scale from 1 to 7, where a score of 1 = "No Benefits" and 7 = "Substantial Benefits". Select "Don't Know" if you're not familiar enough with the given practice to estimate its benefits.

3) To what extent does implementing each of the following practices improve general environmental quality? Examples of environmental benefits might include, but are not limited to, improvements in water quality, water-use efficiency, wildlife habitat, and soil erosion control. For each practice, select your answer on a scale from 1 to 7, where a score of 1 = "No Benefits" and 7 = "Substantial Benefits". Select "Don't Know" if you're not familiar enough with the practice to estimate its benefits.

Water and Soil Management

	1) Economic expense	2) Economic benefits	3) Environmental benefits
Use ET-based methods to determine when to irrigate			
Rely on visual observations to determine when to irrigate			
Use regulated deficit irrigation (RDI) methods			
Measure soil moisture to track water availability			
Measure plant water stress (e.g., pressure bomb)			
Mapping for soil water holding capacity			

Cal Sust Vit - Post Advisor Comments

Use of vegetative filter strips to reduce runoff into waterways			
Written erosion control plan			
Diversion structures (e.g., straw bales, water bars) to divert or contain seasonal water flows			
Soil tests for nutrient content, pH, electrical conductivity (EC), and toxicity			

Cal Sust Vit - Post Advisor Comments

Costs and Benefits: Other Vineyard and Operation Management Practices

The following questions ask you to estimate the costs and benefits to growers of various practices. When answering, consider the costs and benefits that would accrue to a viticultural operation in the geographic region you have the most experience in, and of the size you have the most experience working with. Assume that the practices are being correctly implemented.

First, read the three questions below. Then, for each practice, select an answer to each corresponding question using the dropdown menus.

1) How expensive is it to implement each of the following practices? For each practice, please select your answer on a scale from 1 to 7, where a score of 1 = "Very Inexpensive" and 7 = "Very Expensive". Select "Don't Know" if you're not familiar enough with the practice to accurately estimate its costs.

2) To what extent does implementing each of the following practices produce economic benefits to the grower? Examples of economic benefits might include, but are not limited to, improvements in winegrape quality, reductions in input costs, and improved vineyard health. For each practice, please select your answer on a scale from 1 to 7, where a score of 1 = "No Benefits" and 7 = "Substantial Benefits". Select "Don’t Know" if you’re not familiar enough with the given practice to estimate its benefits.

3) To what extent does implementing each of the following practices improve general environmental quality? Examples of environmental benefits might include, but are not limited to, improvements in water quality, water-use efficiency, wildlife habitat, and soil erosion control. For each practice, select your answer on a scale from 1 to 7, where a score of 1 = "No Benefits" and 7 = "Substantial Benefits". Select "Don’t Know" if you’re not familiar enough with the practice to estimate its benefits.

Other Vineyard and Operation Management Practices

	1) Economic expense	2) Economic benefits	3) Environmental benefits
Use of compost in vineyards			
Owl boxes/perches for birds of prey			
Development of a written company "sustainability" plan			
Develop a written human resource plan			
Develop a written plan to transition the operation to the next generation, or a "succession" plan			
Vineyard management to achieve overall "vine balance"			

Cal Sust Vit - Post Advisor Comments

Monitor and record canopy microclimate throughout growing season			
Third-party certification for "sustainable" or "green" viticulture			
Use of alternative electricity sources such as wind or solar			
Use of alternative fuels such as bio-diesel, propane, natural gas, or methane			
Disposal of removed vines by means other than burning			
Total energy (fuel and electricity) is monitored and recorded			
Primarily use mechanical methods for major viticultural activities (e.g., pruning, harvesting)			

Cal Sust Vit - Post Advisor Comments

Sustainable Viticulture

What does sustainability mean to you in the context of winegrape growing? (Type your answer in the box below.)

5

6

Cal Sust Vit - Post Advisor Comments

Sustainable Viticulture Outreach and Education Programs

Some regions in California have established sustainable viticulture outreach and education programs or partnerships that have the goal of increasing the sustainability of viticulture and winemaking. The sustainable viticulture programs usually seek to increase grower adoption of sustainable practices and promote cooperation among multiple organizations involved with viticulture in a region. Examples include but are not limited to the Central Coast Vineyard Team, the Napa Valley Grapegrowers Association, and the Lodi Winegrape Commission's Sustainable Viticulture Program. We would like your opinion about any sustainable viticulture programs that may exist in your region. If you do not know of any viticulture programs in your region, click "Next" at the bottom of the page.

Are you aware of any sustainable viticulture programs in your region? If yes, please list the name of the program/s:

Program Name

Program Name

Program Name

Program Name

Program Name

Of the programs you listed above, which are you the most familiar with? (List only one in the box below.)

Cal Sust Vit - Post Advisor Comments

For the sustainable viticulture program that you are most familiar with, how successful has it been in achieving the following goals? Please select your answer on a scale from 1 to 7, where a score of 1 = "No Success" and 7 = "Very Successful". Select "NA" if the listed goal is not actually a goal of the program. Select "Don't Know" if you don't have enough information to accurately answer the question.

	No Success	2	3	4	5	6	Very Successful	Don't Know
Improve consumer perceptions of region								
Secure funding to support program activities								
Improve marketability of winegrapes from the region								
Increase trust among growers								
Facilitate cooperation among viticultural organizations								
Increase economic return to wineries using sustainable winegrapes								
Increase economic returns to growers								
Reduce environmental risks								
Promote the adoption of sustainable practices								
Increase grower connection to local community								
Improve social welfare of farm workers								
Improve relationships between growers and wineries								
Increase communication among growers								
Increase capacity of growers to efficiently manage vineyards								
Increase availability of wildlife habitat								
Improve relationship between viticulture industry and regulatory agencies								
Increase level of winery participation in sustainability program								
Increase level of winegrape grower participation in sustainability program								

Cal Sust Vit - Post Advisor Comments

To what extent do the following approaches to winegrape growing and management promote sustainability in general? Please select your answer on a scale from 1 to 7, where a score of 1 = "Does not promote sustainability" and 7 = "Strongly promotes sustainability". Select "Don't Know" if appropriate.

	Does not promote sustainability	2	3	4	5	6	Strongly promotes sustainability	Don't Know
Use of self-assessment sustainable viticulture workbooks								
Sustainable third-party certification								
Organic third-party certification								
Biodynamic								

Cal Sust Vit - Post Advisor Comments

In developing this survey, our research team used existing records to identify as many viticulture outreach, education, and advisor professionals as possible. However, these records often do not identify all relevant individuals. Could you provide us with the names and emails of any other viticulture experts who you think should receive this survey? This information remains confidential in the hands of the researchers and will not be made public.

Names of other possible participants

Name

Name

Name

Name

Name

Emails of other potential participants

Email

Email

Email

Email

Email

Cal Sust Vit - Post Advisor Comments

Thank you for completing this survey about viticulture in California. If you would like to receive a copy of any reports that are generated as a result of this research, please click "Yes" below.

- Yes, I would like to receive copies of reports generated by this research.
- No, I would not like to receive any further information.

Do you have any comments about this survey? (Type your text in the box below.)

Please enter your email address in the box below in order to confirm completion of the survey, and to ensure that you will not receive any additional reminders about this survey. (Data from the survey will be kept anonymous and we will not use your email for any other purpose.)

Please contact Mark Lubell if you have any additional questions about this research.

Mark Lubell, Ph.D.
Department of Environmental Science and Policy
University of California, Davis
vitresearch@ucdavis.edu
530-752-5880
